# Potential Utility of Natural Killer Cells for Eliminating Cells Harboring Reactivated Latent HIV-1 Following the Removal of CD8^+^ T Cell-Mediated Pro-Latency Effect(s)

**DOI:** 10.3390/v13081451

**Published:** 2021-07-26

**Authors:** Georges Khoury, Deanna A. Kulpa, Matthew S. Parsons

**Affiliations:** 1 Division of Microbiology and Immunology, Yerkes National Primate Research Center, Emory University, Atlanta, GA 30322, USA; georges.khoury@emory.edu (G.K.); deanna.kulpa@emory.edu (D.A.K.); 2 Department of Pathology and Laboratory Medicine, Emory University School of Medicine, Atlanta, GA 30322, USA

**Keywords:** NK cells, HIV-1 latency, HIV-1 cure, CD8^+^ T cells

## Abstract

An impediment to curing HIV-1 infection is the persistence of latently infected cells in ART-treated people living with HIV (PLWH). A key strategy for curing HIV-1 infection is to activate transcription and translation of latent virus using latency reversing agents (LRAs) and eliminate cells harboring reactivated virus via viral cytopathic effect or immune clearance. In this review, we provide an overview of available LRAs and their use in clinical trials. Furthermore, we describe recent data suggesting that CD8^+^ T cells promote HIV-1 latency in the context of ART, even in the presence of LRAs, which might at least partially explain the clinical inefficiency of previous “shock and kill” trials. Here, we propose a novel cure strategy called “unlock, shock, disarm, and kill”. The general premise of this strategy is to shut down the pro-latency function(s) of CD8^+^ T cells, use LRAs to reverse HIV-1 latency, counteract anti-apoptotic molecules, and engage natural killer (NK) cells to mediate the killing of cells harboring reactivated latent HIV-1.

## 1. Introduction

Despite major advances in the treatment and management of HIV-1 infection, curative strategies remain elusive. Treatment of HIV-1 infection with antiretroviral therapy (ART) arrests viral replication and reduces morbidity and mortality [1,2]. Importantly, ART does not cure HIV-1, as the virus persists in latently infected cells in ART-treated people living with HIV (PLWH) [3,4]. Upon ART interruption, latent virus is responsible for the rapid rebound of virus replication [5,6]. Eliminating cellular sources of latent HIV-1 could cure HIV-1 infection and liberate infected individuals from needing lifelong ART to suppress viral replication.

Efforts to design a cure for HIV-1 have primarily focused on the “shock and kill” approach, which proposes pharmacological intervention to awaken HIV-1 from latency and the elimination of cells carrying reactivated virus via viral cytopathic effect or immune clearance [7,8]. This strategy should be performed in the context of ART to prevent viral spread to new cellular targets. Several pharmacological latency reversing agents (LRAs) have been identified and demonstrated to reverse HIV-1 latency [9]. A subset of these LRAs have been assessed for their ability to reactivate latent HIV-1 in vivo in pre-clinical animal studies and human clinical trials [10,11,12,13,14,15,16,17,18,19,20,21,22,23,24]. In general, the administration of LRAs to ART-treated PLWH has produced only modest reactivation of latent virus and has had little to no impact on the size of the HIV-1 reservoir. These studies have motivated efforts to identify more potent LRAs, which reactivate virus more robustly in vivo and facilitate immune recognition and elimination of cellular sources of latent virus. A potential alternative strategy to designing new LRAs is to improve the in vivo activity of currently available LRAs by modulating the capacity for immune cells to sustain latency in their presence.

Recent studies have demonstrated that CD8^+^ lymphocytes play a role in sustaining HIV-1 latency in the context of ART [25], and even in the presence of LRAs [22,23]. This phenomenon has been observed in vivo in ART-treated SIV- or SHIV-infected rhesus macaques and ART-treated HIV-1-infected humanized mice [22,23,25]. It has also been recapitulated in vitro using human cells [22]. Although the exact identity of the CD8^+^ lymphocyte population responsible for promoting viral latency remains unresolved, evidence from the rhesus macaque model suggests that CD8^+^ T cells contribute to the maintenance of viral latency, but CD8^+^ natural killer (NK) cells are not involved [25]. This is an important observation, as NK cells can kill HIV-1-infected cells via both direct and antibody-dependent mechanisms [26,27,28,29]. Collectively, these observations provide a rationale for designing an improved “shock and kill” strategy, whereby pro-latency CD8^+^ T cell function(s) is inhibited, LRAs are administered, anti-apoptotic molecules are counteracted, and NK cells are engaged to eliminate cells harboring reactivated latent HIV-1.

Here, we discuss the current LRA landscape and review completed pre-clinical and clinical trials of the “shock and kill” approach. We recount the evidence that CD8^+^ T cells contribute to the maintenance of HIV-1 latency in the context of ART and in the presence of LRAs. Finally, we examine a potential multi-pronged HIV-1 cure approach that employs NK cells as cytolytic effector cells.

## 2. The LRA Landscape

**Figure 1:**
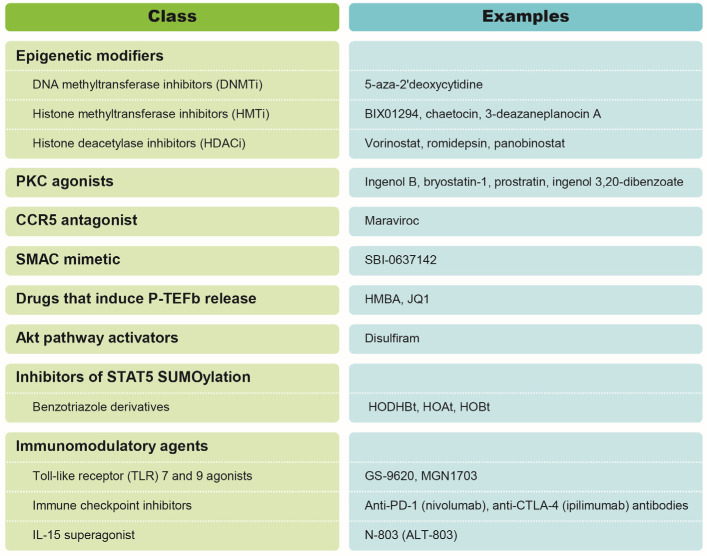
HIV-1 latency reversing agents.

Various classes of HIV-1 LRAs have been identified for their ability to induce reactivation of latent HIV-1. These agents have been assessed using in vitro (i.e., latently infected cell lines and primary cell models of latency) and ex vivo (i.e., reactivation of virus from cells derived from ART-treated PLWH) cell culture systems. The LRAs reviewed in this section are summarized in Figure 1.

A major class of LRAs are epigenetic modifiers, such as DNA methyltransferase inhibitors (DNMTi), histone methyltransferase inhibitors (HMTi), and histone deacetylase inhibitors (HDACi). During latency, nucleosomes surrounding the HIV-1 5′long terminal repeat (5′LTR) are suppressed by epigenetic modifications, such as DNA methylation of the two CpG islands surrounding the viral transcription start site, histone di- and tri-methylation (H3K9me2, H3K9me3, and H3K27me3), and deacetylation, induced by DNA methyltransferases (DNMT), histone methyltransferases (HMT), and histone deacetylases (HDAC), respectively [30,31]. Treatment of latently infected cells with DNMTi (e.g., 5-aza-2′deoxycytidine), HMTi (e.g., BIX01294, chaetocin, and 3-deazaneplanocin A), or HDACi (e.g., vorinostat, romidepsin, and panobinostat) induces chromatin decondensation and relieves transcriptional repression at the 5′LTR resulting in HIV-1 expression [32,33,34,35,36,37,38,39,40,41].

Protein kinase C (PKC) agonists also exhibit LRA activity. Indeed, PKC agonists, such as ingenol B [42], bryostatin-1 [43,44], prostratin [45], and ingenol 3,20-dibenzoate [46] disrupt HIV-1 transcriptional repression in latently infected cells and induce virus production. The capacity of PKC agonists to reverse HIV-1 latency is linked to their ability to activate NF-κB [9,31]. Activation of NF-κB is a frequent target for LRAs, including the CCR5 antagonist maraviroc, as well as second mitochondria-derived activator of caspases (SMAC) mimetics, which activate NF-κB through the noncanonical pathway [9,21,47,48].

Drugs that induce positive transcription elongation factor b (p-TEFb) release have also been investigated as LRAs. HIV-1 reactivation has been achieved through the use of molecules, such as HMBA, that induce p-TEFb release from inactive complexes [49] or drugs that inhibit bromodomain 4 (BRD4) [50], a cellular protein that competes with the viral Tat protein for binding to p-TEFb. Drugs that induce p-TEFb release have also been shown to act in synergy with other LRAs to induce viral reactivation in in vitro HIV-1 latency models [51]. In ex vivo cultures of resting CD4^+^ T cells from ART-treated PLWH, combinations of the bromodomain and extra-terminal domain inhibitor (BETi) JQ1 with PKC agonists (i.e., JQ1 + bryostatin-1 and JQ1 + ingenol-B) potently reactivated latent HIV-1 and induced the production of similar levels of virus as stimulation with anti-CD3 and anti-CD28 antibodies [51].

Another class of LRAs are activators of the Akt signaling pathway, such as disulfiram, a drug used for the treatment of alcohol use disorder. Disulfiram has been shown to reactivate HIV-1 in latently infected cell lines of myeloid but not T-lymphoid origin [52,53]. The drug also shows LRA activity in a primary BCL-2-transduced CD4^+^ T cell model of latency, without inducing global T cell activation [54].

Another group of LRAs that promote HIV-1 reactivation are benzotriazole derivatives, such as 3-hydroxy-1,2,3-benzotriazin-4(3H)-one (HODHBt), 1-hydroxybenzotriazol (HOBt), and 1-hydroxy-7-amino benzotriazole (HOAt). HODHBt has been shown to reactivate latent HIV-1 in the presence of interleukin-2 (IL-2) or interleukin-15 (IL-15) [55,56]. This class of LRAs impairs signal transducer and activator of transcription 5 (STAT5) SUMOylation and increases STAT5 activity and occupancy of the HIV-1 5′LTR [55].

Lastly, several immunomodulatory agents have been shown to exhibit LRA activity. These agents include Toll-like receptor (TLR) 7 and 9 agonists (i.e., GS-9620 and MGN1703) [57,58] and immune checkpoint inhibitors, such as anti-CTLA-4 and anti-programmed cell death 1 (PD-1) monoclonal antibodies [59,60]. Lastly, the IL-15 superagonist N-803 (also known as ALT-803) induces HIV-1 transcription in both an in vitro primary cell model of latency and ex vivo in cells derived from PLWH [61].

A monumental amount of work has been performed to characterize the LRA activity of the agents discussed above. It should be noted, however, that currently available LRAs reactivate only a small portion of the reservoir, with estimates ranging from <5% (in vitro primary cell model using reporter virus) to 3–31% (ex vivo activation of cells from PLWH) [62,63]. Furthermore, the activity of LRAs in in vitro models does not always reflect their activity ex vivo. A comparison of LRA activity across five in vitro primary T cell models, four J-Lat cell line models, and resting CD4^+^ T cells derived from ART-treated PLWH revealed that none of the in vitro models were able to truly recapitulate the ex vivo activity of LRAs on latently infected cells isolated from HIV-1-infected individuals on ART [64]. It is clear, however, from ex vivo experiments, that treatment with a combination of LRAs may be required to achieve an adequate amount of virus reactivation to facilitate efforts to diminish the size of the latent reservoir [65]. More research is urgently needed to discover novel LRA targets or strategies to enhance the efficacy of currently available LRAs.

## 3. Clinical Trials of the “Shock and Kill” Approach

The ability of LRAs to reactivate latent HIV-1 has mainly been examined in cell lines or primary cell models of latency, and only a limited number of LRAs have been assessed in clinical trials. In these clinical trials, LRAs have been investigated in ART-treated PLWH for their potential to initiate HIV-1 transcription, measured as an increase in cell-associated unspliced (US) HIV-1 RNA, and reactivate virus production, measured as an increase in plasma HIV-1 RNA. The HDACi vorinostat was selected for testing in clinical trials for its ability to reverse HIV-1 latency in ART-treated PLWH, due to its prior FDA approval for use as a treatment for cutaneous T cell lymphoma [66]. In the context of ART-treated HIV-1 infection, a single 400 mg oral dose of vorinostat induced an increase in cell-associated US HIV-1 RNA (mean 4.8-fold) in circulating resting CD4^+^ T cells (NCT01319383) [20]. However, no significant change in plasma HIV-1 RNA was observed. A subsequent clinical trial tested daily administration of 400 mg of vorinostat for 14 days in ART-treated PLWH and also observed an increase in cell-associated US RNA (NCT01365065) [12]. Panobinostat, another HDACi, was also evaluated in a phase 1/2 clinical trial (NCT01680094) that included 15 HIV-1-infected participants on ART. Oral administration of 20 mg panobinostat three times per week, every other week for 8 weeks, increased cell-associated US HIV-1 RNA (median maximum increase of 3.5-fold) and plasma viremia [14]. However, no cohort-wide reduction in the size of the latent reservoir was observed following panobinostat administration. The HDACi romidepsin was also evaluated in vivo in a proof-of-concept phase Ib/IIa trial (NCT02092116) [16]. Intravenous romidepsin administration (5 mg/m^2^) once weekly for three weeks while on ART led to an increase in both cell-associated US RNA and plasma HIV-1 RNA. Despite its latency reversing effect, no cohort-wide decrease in the reservoir size was observed following romidepsin administration.

The reactivation of latent HIV-1 was also assessed by single administration of PKC agonist bryostatin-1 (10 or 20 µg/m^2^) in a double-blind phase I clinical trial (NCT02269605). No change in cell-associated US HIV-1 RNA was observed, potentially due to low bryostatin-1 plasma concentrations [13].

Disulfiram has also been tested clinically as an LRA [11]. A prospective dose escalation study (NCT01944371) showed that daily dosing of disulfiram at 500, 1000, or 2000 mg/day was safe and well tolerated in ART-treated PLWH. Furthermore, all doses of the drug induced an increase in HIV-1 cell-associated US RNA. When disulfiram was administered at the highest dose, an increase in plasma HIV-1 RNA was noted. However, disulfiram was shown to be ineffective in reducing the size of the latent reservoir.

TLR agonists have also been tested for their ability to reverse viral latency in vivo in ART-treated PLWH and ART-treated SHIV-infected nonhuman primates. Subcutaneous administration of TLR-9 agonist MGN1703 (60 mg), twice weekly for four weeks, in virologically suppressed PLWH on ART (NCT02443935) increased plasma HIV-1 RNA (6/15 participants) but did not change the levels of total or integrated DNA [17]. In a follow-up study, ART-treated PLWH were dosed with MGN1703 for 24 weeks and then underwent ART interruption [18]. A proportion of the PLWH stopped both ART and MGN1703, while others stopped ART and continued receiving MGN1703. Both sets of PLWH experienced viral rebound at a median of 14 days after ART interruption. The authors noted that the time to rebound was similar to previous studies of ART interruption. Borducchi et al. showed a delay in viral rebound following ART interruption in SHIV_SF162P3_ infected rhesus macaques after the administration of the broadly neutralizing antibody PGT121 in combination with TLR-7 agonist GS-9620 [10]. The authors suggested GS-9620 may have activated latently infected CD4^+^ T cells, rendering them more susceptible to antibody-dependent NK cell elimination. In a recent multicenter, double-blind, placebo-controlled, dose escalation (1–12 mg) trial (NCT02858401), GS-9620 was shown to induce immune activation in virally suppressed PLWH. However, no changes in the levels of cell-associated HIV-1 RNA or DNA, or plasma viremia, were noted [15].

Lastly, IL-15 superagonist N-803 has been tested in the pre-clinical ART-treated SHIV-infected rhesus macaque model for in vivo LRA activity [24]. Despite its ability to reactivate latent virus in vitro and ex vivo [61], N-803 did not reverse latency in ART-treated SHIV-infected macaques [24]. Studies from the laboratory of Dr. Guido Silvestri suggest that in vivo LRA activity of agents such as N-803 might be attenuated by the capacity of CD8^+^ T cells to promote the maintenance of viral latency [22,23].

## 4. Evidence That CD8^+^ T Cells Promote HIV-1, SIV, and SHIV Latency during ART and in the Presence of LRAs

### 4.1. CD8^+^ T Cells in HIV-1, SIV, and SHIV Infections

Much evidence implicates CD8^+^ T cells in the in vivo control of HIV-1 replication in untreated PLWH and SIV and SHIV replication in untreated nonhuman primates. First, the development of anti-viral CD8^+^ cytotoxic T lymphocyte (CTL) responses corresponds with the initial control of viral replication during primary HIV-1 infection [67,68]. Second, anti-viral CD8^+^ CTLs drive the generation of viral escape mutants [69,70]. Third, anti-viral CD8^+^ T cells restricted to particular major histocompatibility complex class I (MHC-I) molecules associate with the control of viral replication [71]. Fourth, in vivo depletion of CD8^+^ T cells in SIV- or SHIV-infected rhesus macaques with untreated infections increases viral replication [72,73,74,75].

Although it is well established that CD8^+^ T cells contribute to controlling HIV-1 infection, the relative importance of individual CD8^+^ T cell-mediated functions remains unclear. Anti-HIV-1 CD8^+^ T cells contribute to controlling HIV-1 viremia through the cytolysis of infected cells [76]. CD8^+^ T cells also contribute to the control of HIV-1 replication via non-cytolytic mechanisms. These mechanisms include the production of beta chemokines (i.e., RANTES, MIP-1α, and MIP-1β), which bind and occlude C-C chemokine receptor 5 (CCR5), preventing CCR5 tropic HIV-1 strains from accessing co-receptor and blocking the process of viral entry [77,78]. Furthermore, CD8^+^ T cells can inhibit the replication of CXCR4 tropic viruses through the release of MDC, TARC, I-309, angiogenin, and RNase 4 [79]. Lastly, CD8^+^ T cells are able to inhibit HIV-1 transcription through the release of an unidentified soluble factor, which has been termed CD8^+^ cell antiviral factor (CAF) [80,81,82,83,84,85,86,87].

Recently, a series of studies provided evidence that CD8^+^ T cells inhibit viral transcription in the context of ART and interfere with efforts to awaken latent HIV-1 through the administration of LRAs [22,23,25]. Understanding the mechanism(s) of this pro-latency activity of CD8^+^ T cells will facilitate the design of novel HIV-1 cure strategies. In the following sections, we review the evidence that CD8^+^ T cells mediate pro-latency function(s) during ART and following the administration of LRAs. We also discuss what is currently known about the mechanism(s) of CD8^+^ T cell-mediated pro-latency function(s). Finally, we examine outstanding questions about the role of another lymphocyte population, NK cells, in promoting viral latency.

### 4.2. Impact of CD8^+^ T Cell Depletion on Viremia in a Nonhuman Primate Model of ART-Treated HIV-1 Infection

The initial evidence that CD8^+^ T cells promote the control of HIV-1 replication in the context of ART was derived from a study by Cartwright et al. that assessed the impact of CD8^+^ leukocyte depletion in ART-treated SIV-infected rhesus macaques [25]. Thirteen macaques were intravenously infected with SIV_mac239_ and at eight weeks post-infection were placed on an ART regimen for 8–32 weeks consisting of tenofovir, emtricitabine, raltegravir, and darunavir prior to further intervention. During this period, plasma viremia declined >99% from the pre-ART period, with 12/13 animals achieving plasma viremia <60 copies/mL. Seven animals exhibited a pattern of consistent viral suppression (i.e., at least four consecutive undetectable viremia measurements of <60 copies/mL), and five animals exhibited intermittent viral suppression (i.e., mixtures of undetectable and detectable plasma viremia). The remaining animal experienced an ART-driven decrease in plasma viremia of more than 5 logs but never reached undetectable. At times corresponding to consistent viral suppression or three non-consecutive plasma viremia readings of <60 copies/mL, animals were intravenously administered a single dose of the simianized anti-CD8α MT-807R1 antibody (50 mg/kg) to deplete CD8^+^ leukocytes. The animal that never achieved full viral suppression at any time was administered MT-807R1 at 32 weeks following the initiation of ART. Administration of MT-807R1 robustly depleted >95% of CD8^+^ T cells in the periphery at one day post-infusion. Depletion was less robust in lymph nodes and rectal tissue, with 70% and 62% depletion noted at one week following MT-807R1 infusion. Notably, as macaque NK cells express CD8α, a robust depletion of NK cells was also observed in the periphery. Intriguingly, despite maintaining ART throughout the CD8 depletion protocol, all 13 animals experienced an increase in SIV viremia following CD8 depletion (72- to 350-fold—as determined by analyzing a subset of animals). Plasma viremia occurred between one day and three weeks after CD8 depletion. Using RNAscope technology, it was revealed that CD8 depletion also resulted in increased numbers of SIV RNA producing cells within the lymph nodes of ART-treated SIV-infected macaques.

Given that the infused MT-807R1 antibody depleted both CD8^+^ T cells and NK cells, the authors performed analyses to determine the cell subset(s) driving post-depletion viral rebound [25]. It was noted that the post-depletion repopulation dynamics of CD8^+^ T cells and NK cells were distinct from one another. Although an increase in viremia corresponded with the initial depletion of NK cells, this viremia was not controlled by the repopulation of NK cells. Viral rebound in ART-treated SIV-infected CD8α^+^ leukocyte depleted macaques was temporally linked to the removal of CD8^+^ T cells, and reestablishment of viral control corresponded to the repopulation of CD8^+^ T cells.

In the final component of the Cartwright et al. study, the investigators attempted to identify the mechanisms promoting the viremia observed following CD8 depletion and the origins of the rebounding virus [25]. While CD8 depletion was coupled with homeostatic proliferation of CD4^+^ T cells, the authors observed no correlation between CD4^+^ T cell proliferation and viral rebound. However, HLA-DR (PBMC and rectal biopsies) and PD-1 (PBMC) expression on CD4^+^ T cells was correlated with post-CD8 depletion viremia at early time points, suggesting that CD4^+^ T cell activation following CD8^+^ T cell removal could be a contributing factor to post-depletion viremia. Finally, in an attempt to identify the source of the rebounding virus, the authors noted that the virus that rebounded was most similar in sequence to the virus present during primary infection. This suggests that the rebounding virus was produced from long-lived cells infected prior to ART initiation.

The data summarized above clearly illustrate an important role for CD8^+^ T cells in controlling virus production during ART. From this initial study [25], it was unclear if the rebound in viremia following CD8^+^ T cell depletion was due to a reversal of viral latency or an amplification of ongoing low-level viral replication. Nevertheless, the observation of detectable viremia during ART in CD8^+^ T cell depleted animals led to follow-up studies assessing if coupling CD8^+^ T cell depletion with LRA administration would enhance the in vivo efficacy of LRAs and facilitate the design of novel HIV-1 eradication strategies.

### 4.3. Impact of CD8^+^ T Cell Depletion on In Vivo LRA Activity in Animal Models of ART-Treated HIV-1 Infection

The Cartwright et al. study provided data implicating CD8^+^ T cells in the inhibition of HIV-1 viral production during ART [25]. One possible explanation for the reported observations is that CD8^+^ T cells promote the maintenance of viral latency. A ramification of this possibility is that LRA administration in the absence of CD8^+^ T cells should increase the in vivo efficacy of LRAs. In a series of follow-up studies, McBrien et al. tested the in vivo latency reversing activity of the IL-15 superagonist N-803 in ART-treated SIV- or SHIV-infected rhesus macaques and ART-treated HIV-1-infected humanized mice that were CD8^+^ T cell competent or depleted [22,23]. The N-803 IL-15 superagonist is a fusion protein consisting of a mutant IL-15 (IL-15 N72D) linked to a dimeric IL-15 receptor αSu and human IgG_1_ Fc fusion protein [88]. Compared to soluble IL-15, this complex exhibits higher biological activity and a longer serum half-life [88,89]. Finally, N-803 has been shown to reverse HIV-1 latency in in vitro and ex vivo experimental systems [61].

McBrien et al. first tested the in vivo LRA activity of N-803 in the presence or absence of CD8^+^ T cells using the ART-treated SIV-infected rhesus macaque model [22]. Thirty-five macaques were intravenously infected with SIV_mac239_ and initiated on an ART regimen consisting of tenofovir, emtricitabine, and dolutegravir starting at 56 days post-infection. All animals were maintained on ART for at least one year prior to additional interventions. During ART, all animals achieved undetectable plasma viremia (i.e., <60 copies/mL). At the time of experimental intervention, 33/35 animals had undetectable plasma viremia. For the experimental component of the study, the animals were divided into three groups. The first group consisted of seven animals that were subcutaneously treated once a week for four weeks with N-803 (100 µg/kg). The second group consisted of 14 animals that were administered one dose of the anti-CD8α antibody MT-807R1 to deplete CD8^+^ leukocytes. The third group consisted of 14 animals that underwent CD8 depletion and were given four weekly doses of N-803 starting at the time of MT-807R1 administration.

Analyses of the experimental groups revealed that treatment of CD8^+^ leukocyte competent ART-treated SIV-infected macaques with N-803 did not increase plasma viremia to >60 copies/mL in any of the tested animals [22]. Consistent with the Cartwright et al. study [25], 11/14 animals solely depleted of CD8^+^ leukocytes exhibited plasma viremia > 60 copies/mL [22]. Viremia > 60 copies/mL was detected in 32.1% of the collected samples, and viremia > 1000 was noted in 2/14 animals and in 3.6% of the collected samples. The appearance of viremia was temporally linked to CD8^+^ T cell depletion, and the control of viremia was re-established upon CD8^+^ T cell repopulation. Finally, in the third group of animals that were both depleted of CD8^+^ leukocytes and administered four weekly doses of N-803, a more robust increase in plasma viremia was observed. Plasma viremia > 60 copies/mL was recorded in 14/14 animals and in 73.2% of collected samples. Plasma viremia > 1000 copies/mL was also observed in 6/14 animals and in 23.2% of collected samples. As with the animals solely depleted of CD8^+^ leukocytes, the control of viremia was re-established upon CD8^+^ T cell repopulation.

The data from the three groups of ART-treated SIV-infected rhesus macaques are consistent with the notion that CD8^+^ T cells promote the maintenance of HIV-1 latency during ART and inhibit the latency reversing activity of LRAs. To further probe the source of the rebounding virus, McBrien et al. performed a sequence analysis on virus present during acute infection, immediately preceding ART and during peak viremia subsequent to CD8^+^ leukocyte depletion in CD8 depleted N-803 treated macaques [22]. The authors noted that the virus present during acute infection was highly homogenous and similar to the challenge stock, while the virus present in the pre-ART and post-CD8 depletion samples was more diverse. The virus present post-CD8 depletion appeared to reflect a broad reactivation of latent virus, as there was no evidence of ongoing viral replication. Indeed, the virus present post-CD8 depletion exhibited no evidence of viral evolution. Furthermore, the animals depleted of CD8^+^ leukocytes and dosed with N-803 did not have an increase in two-long-terminal repeat (2-LTR) circles: a marker of recent infection.

In an effort to confirm that the pro-latency function of CD8^+^ T cells observed in ART-treated SIV-infected macaques extended across animal models, McBrien et al. performed N-803 administration, CD8 depletion, or CD8 depletion plus N-803 administration in ART-treated HIV-1-infected humanized mice [22]. The authors infected bone marrow-liver-thymus (BLT) humanized mice with HIV-1_JR-CSF_ and suppressed viral replication using an oral ART regimen consisting of emtricitabine, tenofovir, and raltegravir four-five weeks after infection. Viral loads were assessed using an assay with a limit of detection of 346 viral RNA copies per mL of plasma. The animals were treated until virally suppressed for four weeks and were then dosed with a single treatment of the N-803 IL-15 superagonist, MT-807R1 anti-CD8α antibody, or a combination of MT-807R1 and N-803 while being maintained on ART. Similar to the results obtained using the ART-treated SIV-infected rhesus macaque model, no viral rebound was observed in the seven mice only administered N-803. A modest level of viral rebound was noted in 3/8 mice solely depleted of CD8^+^ leukocytes. Finally, viral rebound was noted in 7/8 mice both depleted of CD8^+^ leukocytes and administered N-803. These data again suggest that the latency reversal potential of N-803 is impeded by pro-latency function(s) mediated by CD8^+^ T cells.

Finally, in a set of experiments published across two papers, McBrien et al. assessed the pro-latency function(s) of CD8^+^ T cells in five SHIV_SF162P3_-infected rhesus macaques [22,23]. For the first set of experiments, the five macaques were infected with SHIV_SF162P3_ and placed on an ART regimen consisting of tenofovir, emtricitabine, and dolutegravir 12 weeks post-infection. The ART was maintained for six months and then all animals were depleted of CD8^+^ leukocytes using the MT-807R1 anti-CD8α antibody and were administered four weekly doses of N-803, while being maintained on ART. Using an ultrasensitive PCR assay capable of detecting three copies of viral RNA per mL of plasma, the authors reported that the intervention reactivated SHIV in all five animals.

In a follow-up study, McBrien et al. utilized the same ART-treated SHIV_SF162P3_-infected animals to assess whether depleting CD8^+^ T cells without simultaneously depleting NK cells produced a similar virological result following N-803 administration [23]. To achieve this goal, the authors depleted CD8^+^ T cells using a simianized anti-CD8β antibody, CD8b255R1. Macaque CD8^+^ T cells express CD8α as a homodimer or heterodimer in combination with CD8β. Alternatively, macaque NK cells mostly express CD8α as a homodimer [90]. As such, administration of CD8b255R1 depleted CD8^+^ T cells but not NK cells [23]. The depletion obtained using this antibody was less efficient than the depletion observed following MT-807R1 administration. Furthermore, the repopulation of CD8^+^ T cells after CD8b255R1 administration occurred within one week. Nevertheless, depletion of CD8^+^ T cells using CD8b255R1 coupled with N-803 administration resulted in viral rebound in 3/5 animals.

**Figure 2:**
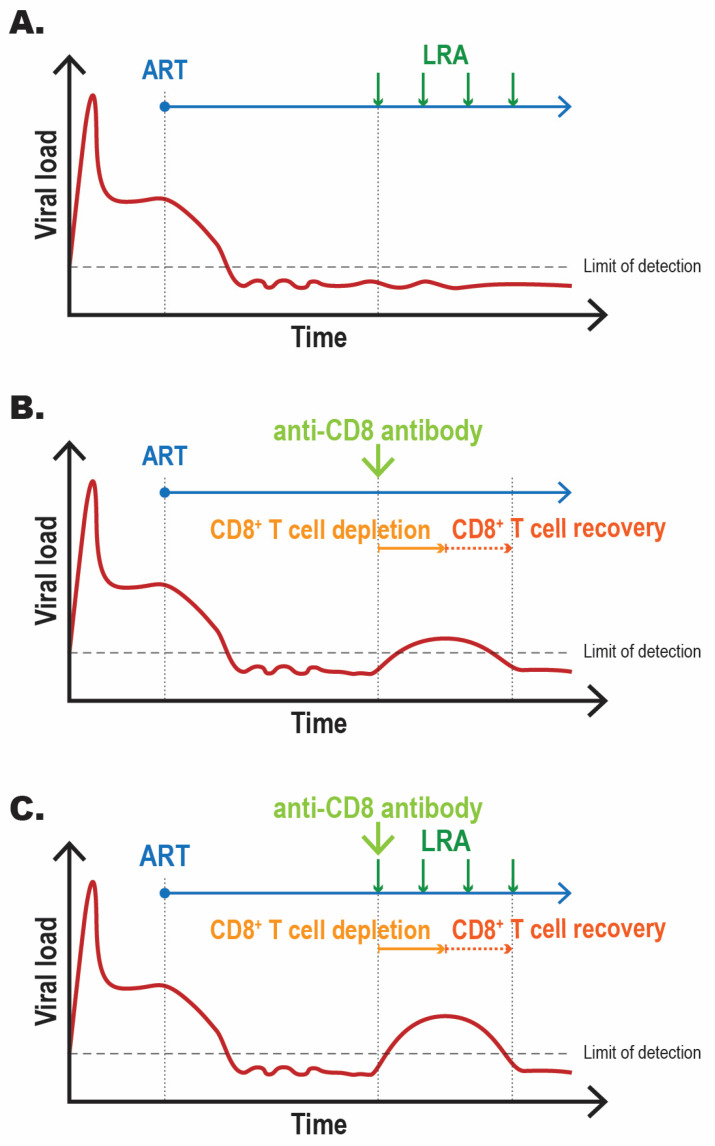
Viral dynamics following LRA administration and/or CD8^+^ T cell depletion in ART-treated SIV-infected rhesus macaques. The three graphs summarize the impact of (**A**) LRA administration, (**B**) CD8^+^ T cell depletion, or (**C**) combined CD8^+^ T cell depletion and LRA administration in ART-treated SIV-infected rhesus macaques. The graphs represent a summary of the general findings reported by McBrien et al. [22]. (**A**) LRA administration does not result in detectable viremia. (**B**,**C**) CD8^+^ T cell depletion results in detectable viremia, which is enhanced if LRA is administered concomitantly. Control of viremia is observed following CD8^+^ T cell repopulation. To facilitate a simple comparison of the three conditions, the graph depicted in (**C**) was prepared under the assumption that the administered LRA does not impact the dynamics of CD8^+^ T cell recovery post-depletion.

The results of the experiments performed by McBrien et al. are summarized in Figure 2 [22]. Collectively, these experiments provide evidence that CD8^+^ T cells suppress viremia during ART by impeding viral transcription and promoting the maintenance of viral latency. This contention is supported by data highlighting that viral rebound following CD8 depletion and N-803 administration does not coincide with viral evolution or an increase in the levels of 2-LTR circles. More work is needed to decipher the mechanism(s) of this pro-latency activity of CD8^+^ T cells to identify a new pathway(s) to target to improve the in vivo efficacy of LRAs and the outcomes of HIV-1 cure strategies.

### 4.4. Deciphering the Mechanism(s) of CD8^+^ T Cell-Mediated Pro-Latency Function(s) Using In Vitro Assays

Previous in vitro studies by the group of Dr. Jay Levy have suggested that CD8^+^ T cells suppress HIV-1 expression via non-cytolytic mechanisms related to the secretion of a soluble factor(s) [81,82,83,85,86,87]. While this putative lymphocyte associated anti-HIV-1 factor, termed CAF, has never been identified, studies have shown that suppression of HIV-1 expression can be mediated by the production of CAF by CD8^+^ T cells lacking specificity for HIV-1 [91], and that CAF suppresses viral gene expression and LTR activation independent of blocking viral entry, integration, or reverse transcription [80,83,84].

To characterize these observations further, Zanoni et al. examined the influence of TCR-activated CD8^+^ T cells from HIV-1 naïve donors on HIV-1 expression in co-cultures with autologous in vitro infected CD4^+^ T cells [92]. The authors observed potent suppression of HIV-1 transcription in CD4^+^ T cells that was both non-cytotoxic and MHC-I independent. The suppression of HIV-1 expression in the CD8^+^/CD4^+^ co-cultures was associated with reduced CD4^+^ T cell activation and proliferation, as well as the promotion of the survival of infected cells [92].

The experiments performed by Zanoni et al. were conducted in the context of suppression of HIV-1 expression during active infection [92]. To characterize the impact of CD8^+^ T cells on the maintenance of HIV-1 latency during ART, McBrien et al. employed an in vitro primary cell model of HIV-1 latency that had been previously developed and optimized, called the latency and reversion assay (LARA) [22,93]. This model had been previously used to assess whether memory CD4^+^ T cell subsets exhibit differential responses to LRAs [93]. To address the impact of CD8^+^ T cells on LRA activity, latently infected memory CD4^+^ T cells generated in LARA were co-cultured alone or with TCR-activated autologous total CD8^+^ T cells while in the presence of strong TCR activation (anti-CD3/CD28), the IL-15 superagonist N-803, or the gamma-c cytokine IL-15 [22]. The presence of autologous CD8^+^ T cells significantly inhibited the latency reversing activity of all three LRAs, as compared to the CD4^+^ T cell monoculture controls. These studies support a role for CD8^+^ T cells in the maintenance of HIV-1 latency. Although these studies were performed using bulk CD8^+^ T cells, additional studies are underway to identify the specific CD8^+^ T cell subpopulation(s) with pro-latency activity.

### 4.5. Outstanding Questions about the Role of NK Cells in Promoting Viral Latency

Two key pieces of evidence suggest that the promotion of HIV-1 latency by CD8^+^ leukocytes is mediated by CD8^+^ T cells but not NK cells. First, the re-establishment of viral control in CD8 depleted ART-treated SIV-infected macaques corresponds with CD8^+^ T cell repopulation and not NK cell repopulation [25]. Second, administration of N-803 to ART-treated SHIV-infected rhesus macaques depleted of CD8^+^ T cells using an anti-CD8β antibody was sufficient to achieve viral reactivation [23]. Here, we discuss potential caveats to the evidence that NK cells are not involved in promoting HIV-1 latency and experimental strategies to ultimately resolve the role NK cells play in promoting viral latency and impeding the utility of LRAs. Confirming that NK cells do not contribute to the pro-latency function(s) of CD8^+^ leukocytes will facilitate the design of novel strategies that employ NK cells to eradicate cellular sources of latent HIV-1.

The first caveat to the existing data suggesting that NK cells are not involved in the promotion of HIV-1 latency pertains to NK cell differentiation. As the in vivo work resolving the relative contribution of CD8^+^ T cells and NK cells to the promotion of viral latency has been performed in rhesus macaques [25], it is important to review the process of NK cell differentiation in this particular species. Rhesus macaque NK cells have previously been characterized as CD3^−^CD8^Bright^CD20^−/Dim^ or CD3^−^HLA-DR^−^NKG2A^+^ [90,94]. Macaque NK cells can be further divided into subsets on the basis of CD56 and CD16 expression, which appears to reflect their stage of differentiation [90,95,96]. These subsets include CD56^+^ NK cells, double negative NK cells, and CD16^+^ NK cells. The CD56^+^ NK cells have been characterized as immature. They express high levels of lymph node homing markers CCR7 and CD62L and exhibit low levels of perforin and granzyme [90,95,96]. CD56^+^ NK cells also express high levels of the TCF7, ETF1, GATA3, and TCF8 transcription factors, as compared to CD16^+^ NK cells [95]. The CD16^+^ NK cells are more mature and are characterized by low levels of lymph node homing markers CCR7 and CD62L, as well as heightened levels of perforin and granzyme [90,95,96]. CD16^+^ NK cells express higher levels of the BATF transcription factor than CD56^+^ NK cells [95]. The double negative NK cells have been characterized as an intermediate between the CD56^+^ and CD16^+^ NK cells. These are likely NK cells in the process of differentiation [95]. In general, CD16^+^ NK cells are the most frequent subset in the periphery, and CD56^+^ NK cells are frequent in lymphoid and mucosal tissues [96]. Given the differences between macaque NK cell subsets, there is potential for each subset to distinctly contribute to the promotion of viral latency.

In the Cartwright et al. study, the role of NK cells in the promotion of SIV latency was ruled out by an analysis showing that the resolution of viral rebound post-CD8 depletion was not temporally linked to the repopulation of NK cells [25]. This analysis did not take into consideration the differentiation stage of the repopulating NK cells. If the returning NK cells were at a distinct differentiation stage as compared to those that were present prior to CD8 depletion, the analysis conducted could have missed an important role for NK cells in promoting viral latency. Additionally, the analysis performed in Cartwright et al. was based on the repopulation of NK cells in the periphery. As viral reactivation appeared to occur in the lymphoid tissue, a potential important role for NK cells in promoting viral latency could have been missed by not assessing the repopulation of these cells and their differentiation status within lymph nodes.

The second caveat to the notion that NK cells do not promote viral latency is provided by the McBrien et al. study that employed CD8β depletion to selectively remove CD8^+^ T cells [23]. Although this study provided evidence that CD8^+^ T cells are involved in the promotion of viral latency, the fact that the viral rebound was smaller and only in a subset of animals presents the possibility that the non-depleted NK cells are contributing to the maintenance of virus in a latent state. Alternatively, it is also possible that the reduced viral reactivation in CD8β depleted animals treated with N-803 is due to the less efficient depletion of CD8^+^ T cells, as compared to CD8α depleted animals.

Due to these caveats, there is a need for further experimentation to definitively rule out a role for NK cells in the promotion of viral latency during ART and in the presence of LRAs. Such data will facilitate progress on designing HIV-1 eradication strategies focused on engaging NK cells to eliminate cellular sources of latent virus. An ideal way to resolve the role NK cells play in promoting HIV-1 latency, during ART and in the presence of LRAs, is to selectively deplete NK cells from ART-treated SIV-infected rhesus macaques and administer the N-803 IL-15 superagonist. Unfortunately, the currently available protocols for depleting NK cells in rhesus macaques would not resolve questions about the role of NK cells in promoting viral latency. While the administration of anti-CD16 antibody depletes most NK cells in circulation, this method would not remove CD16^−^ NK cells that are frequent within lymphoid and mucosal tissues [95,96,97,98,99]. Another means of depleting NK cells is to administer an anti-IL-15 antibody [100]. While this protocol does lead to the depletion of most NK cells, it also transiently depletes effector memory CD4^+^ and CD8^+^ T cells. As such, this strategy would not resolve outstanding questions about the capacity for NK cells to promote the maintenance of HIV-1 latency.

Given the obstacles to using in vivo NK cell depletion to address the role of NK cells in promoting viral latency during ART and in the presence of LRAs, the potential pro-latency function(s) of NK cells should be assessed using the in vitro culture system (i.e., LARA) reviewed in the previous section [22,93]. Briefly, latently infected CD4^+^ T cells could be cultured alone or with autologous bulk NK cells, NK cells at diverse differentiation stages, and NK cells from distinct anatomical locations in the presence of ART and the absence or presence of LRAs. This experimental set up would allow for a clear evaluation of the ability of NK cells to promote the maintenance of latency and impede the activity of LRAs.

## 5. A Novel Multi-Pronged “Shock and Kill” HIV-1 Cure Strategy

Data from studies of CD8 depletion and N-803 administration indicate that there are hurdles to achieving cytolytic removal of cellular sources of latent virus following viral reactivation [22,23,25]. Below, we discuss these hurdles and present a potential strategy for overcoming this problem.

Both Cartwright et al. and McBrien et al. observed reactivation of latent virus in ART-treated SIV-infected animals following depletion of CD8α^+^ leukocytes, and McBrien et al. reported that this reactivation of virus was more robust in animals treated with N-803 [22,25]. Despite experiencing a reactivation of latent virus, the macaques included in these studies did not exhibit evidence of a decrease in the size of the latent viral reservoir. Indeed, viral DNA within peripheral blood CD4^+^ T cells and lymph node cells was not significantly different between samples collected pre- and post-CD8 depletion. Furthermore, animals treated with N-803 alone, solely depleted of CD8^+^ leukocytes or depleted of CD8^+^ leukocytes and given N-803, exhibited similar viral rebound dynamics when subjected to an analytical treatment interruption three weeks after CD8 reconstitution or the final N-803 dose [22]. Collectively, these data suggest that cells harboring reactivated virus are not undergoing viral cytopathic effects. Furthermore, these data provide no evidence of immune-based elimination of cells carrying reactivated virus. One potential explanation for this observation is that the CD8 depletion protocol removed both anti-viral CD8^+^ T cells and NK cells, two lymphocyte subsets with cytolytic potential. It should be noted, however, that in at least a proportion of animals, NK cells repopulated prior to CD8^+^ T cells. Despite viremia persisting throughout NK cell repopulation, and until the time of CD8^+^ T cell repopulation, no decline in the size of the viral reservoir was noted. It remains unclear if the repopulated NK cells did not clear cells harboring reactivated virus due to the evasion of NK cells by the infected cells, an inability of NK cells to kill cells harboring reactivated virus, or repopulating NK cells being at a differentiation stage with low cytotoxic potential.

Interestingly, no decrease in the size of the viral reservoir was observed following depletion of CD8β^+^ T cells in the presence of N-803 in ART-treated SHIV_SF162P3_-infected rhesus macaques [23]. As CD8β depletion does not remove NK cells, these data raise further concerns about the ability of NK cells to clear cells carrying reactivated virus. One potential explanation for NK cells not clearing infected cells in this context lies in the potential role of NK cells in eliminating CD8β^+^ T cells. If NK cells were involved in killing autologous CD8β^+^ T cells following administration of the CD8b255R1 antibody, they might have been refractory to further cytolytic activity due to activation-induced downregulation of activating receptors [101,102,103]. Given that the reactivation of virus was very brief in the CD8β^+^ T cell depleted animals, NK cells with full cytolytic potential might not have been present during the viral rebound period. Alternatively, it is plausible that NK cells are poor mediators of cytolysis of SHIV-infected cells and require additional activating signals to more robustly kill these cells.

Additional activating signals originating from virus infected cells could be provided to NK cells by anti-viral antibodies bound to the surface of the infected cells, which can cross-link the NK cell FcγRIIIa/CD16 receptor. Activation of NK cells through CD16 triggers antibody-dependent cellular cytotoxicity (ADCC) [104]. The autologous anti-viral antibodies present in SIV- or SHIV-infected macaques and PLWH would likely be insufficient for this purpose. In PLWH, the major targets of ADCC antibodies produced during infection are envelope epitopes that are revealed once the envelope is in the CD4-bound confirmation [105]. HIV-1-infected cells evade these antibodies by downregulating cell surface CD4 via the viral Nef and Vpu proteins [106]. Alternatively, antibodies capable of binding the viral envelope in its native trimeric structure could be passively provided to tag cells carrying reactivated latent virus and increase their susceptibility to removal via NK cell-mediated ADCC [26,27].

Another potential obstacle to killing cells harboring reactivated latent virus is that these cells might exhibit resistance to NK cell-mediated cytolysis. Such resistance could be conferred by expression of anti-apoptotic proteins, such as BCL-2. Indeed, antagonism/silencing of BCL-2 can increase ADCC susceptibility of cancer cells [107,108]. In the context of HIV-1, antagonism of BCL-2 has been shown to increase the susceptibility of cells harboring reactivated latent HIV-1 to CD8^+^ T cell-mediated cytolysis [109]. It is thus possible that antagonism of BCL-2 following the removal of the CD8^+^ T cell pro-latency effect(s) would promote NK cell-mediated cytolysis of cells harboring reactivated latent virus. Additionally, the killing of infected cells could be enhanced through the passive provision of anti-viral antibodies capable of binding the native envelope trimer [26,27]. Finally, depending on the intervention required to inhibit the pro-latency activity of CD8^+^ T cells, cytotoxic anti-viral CD8^+^ T cells might be able to contribute to eliminating cells harboring reactivated latent virus. The mechanism of action of BCL-2 inhibitors and their potential utility for HIV-1 cure strategies have been discussed in detail elsewhere [110,111].

**Figure 3:**
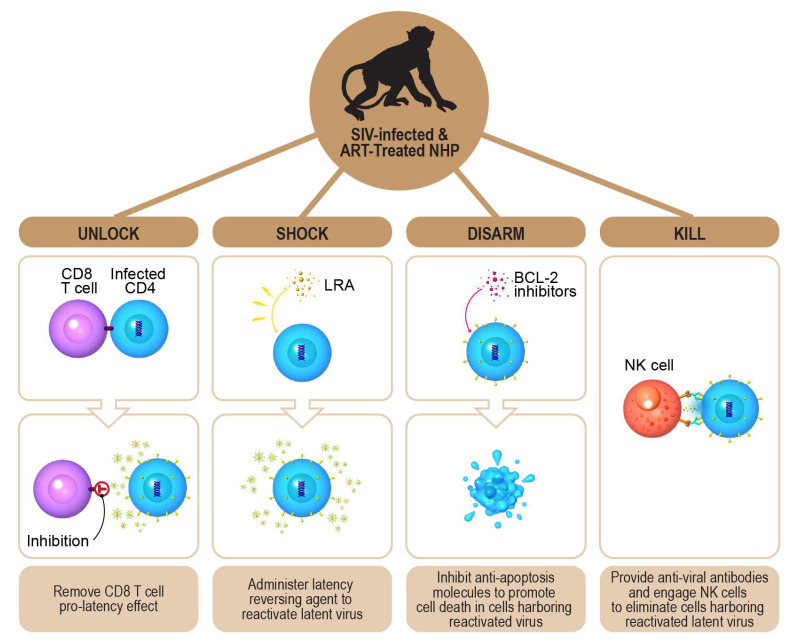
The “unlock, shock, disarm, and kill” strategy for HIV-1 eradication. The diagram depicts the “unlock, shock, disarm, and kill” strategy for HIV-1 eradication in the context of the ART-treated SIV-infected nonhuman primate (NHP) model. This multipronged strategy involves the sequential administration of four interventions. First, ART-treated SIV-infected NHP are administered an agent to inhibit the pro-latency effect(s) of CD8^+^ T cells, which facilitates the reactivation of latent virus in infected CD4^+^ T cells. Second, NHP are treated with pharmacological latency reversing agents (LRA), which further reactivate latent virus. Third, inhibitors of anti-apoptosis molecules are utilized to promote cell death in cells harboring reactivated latent virus. Finally, anti-viral antibodies, which recognize native trimeric envelope spikes, are administered to facilitate the recognition and elimination of cells harboring reactivated latent virus through antibody dependent cellular cytotoxicity (ADCC) mediated by effector cells such as natural killer (NK) cells.

As illustrated in Figure 3, we propose a novel multi-pronged “unlock, shock, disarm, and kill” strategy to advance the goal of HIV-1 eradication. The figure highlights the protocol in the context of the pre-clinical ART-treated SIV-infected nonhuman primate model. First, ART-treated SIV-infected nonhuman primates will be administered an agent to suppress the pro-latency function(s) of CD8^+^ T cells. Second, the animals will be dosed with an LRA capable of broad reactivation of latent virus. Third, a BCL-2 antagonistic agent will be administered. Fourth, ADCC competent anti-viral antibodies capable of recognizing the native envelope trimer on cells harboring reactivated virus will be administered. Finally, the impact of this protocol on the size of the latent viral reservoir will be evaluated.

## 6. Conclusions

A key strategy to cure HIV-1 infection is to administer LRAs, reactivate latent virus, and eliminate cellular sources of latent HIV-1 [7,8]. Numerous pharmacological agents with LRA activity have now been characterized [9]. Although these agents reactivate latent HIV-1 in in vitro assays, their ability to reactivate latent virus following in vivo administration is modest, and they do not facilitate reductions in the size of the reservoir in PLWH [11,12,13,14,15,16,17,18,19,20,21]. Recent evidence suggests that the activity of LRAs can be impeded by CD8^+^ leukocytes, and CD8^+^ T cells appear to be the primary mediator of this pro-latency effect [22,23,25]. Alternatively, NK cells, which can kill HIV-1-infected cells via both direct and antibody-dependent functions [26,27,28,29], do not appear to engage in the promotion of viral latency [25]. We predict that the “unlock, shock, disarm, and kill” strategy, which consists of blocking the pro-latency function(s) of CD8^+^ T cells coupled with LRA administration, would achieve robust reactivation of latent HIV-1 in ART-treated PLWH. Additionally, efforts to block anti-apoptotic molecules and engage cytolytic NK cells under these circumstances could contribute to the goal of eradicating the latent HIV-1 reservoir.

## Data Availability

Not applicable.
